# Activation of Bcl-2-Caspase-9 Apoptosis Pathway in the Testis of Asthmatic Mice

**DOI:** 10.1371/journal.pone.0149353

**Published:** 2016-03-03

**Authors:** Wenyuan Xu, Guifang Guo, Junjuan Li, Zhaolei Ding, Jianhui Sheng, Juan Li, Wei Tan

**Affiliations:** 1 Postgraduate Department of Internal Medicine, Weifang Medical University, Weifang, Shandong, China; 2 Department of Respiratory Medicine, Weifang People's Hospital, Weifang, Shandong, China; University Hospital of Münster, GERMANY

## Abstract

**Background:**

Apoptosis plays a critical role in controlling the proliferation and differentiation of germ cells during spermatogenesis. Dysregulation of the fine-tuned balance may lead to the onset of testicular diseases. In this study, we investigated the activation status of apoptosis pathways in the testicular tissues under the background of an asthmatic mouse model.

**Methods:**

Ten BALB/c mice were divided into two groups: the acute asthma group and the control group. In the acute asthma group, ovalbumin (OVA)-sensitized mice were challenged with aerosolized OVA for 7 days, while the control group was treated with physiological saline. After that, both epididymis and testis were collected to determine the sperm count and motility. Apoptosis in the testis was evaluated by DNA ladder, immunochemistry and further by PCR array of apoptosis-related genes. Finally, the cleavage of caspase-3 and poly ADP-ribose polymerase (PARP) was determined by western blot and the enzymatic activities of caspase-9 and 3/7 were assessed using Caspase-Glo kits.

**Results:**

Compared with control mice, significant decreases in the body weight, testis weight, sperm count and motility were seen in the experimental group. DNA ladder and immunochemistry showed significant increase in apoptotic index of the asthmatic testis, whereas a decrease in mRNA expression of Bcl-2 and increases in Bax, BNIP3, caspase-9, and AIF were observed in the asthma group. Furthermore, protein levels of AIF were significantly upregulated, while the translational expression of Bcl-2 was downregulated markedly. Consistently, caspase-9 activity in the testis of asthma mice was significantly higher than that of the control group.

**Conclusion:**

Collectively, these results showed that Bcl-2-caspase-9 apoptosis pathway was clearly activated in the testis of asthmatic mice with the increased expression of apoptosis-related genes and proteins. To our knowledge, this is the first report demonstrating that asthma could lead to the activation of the mitochondrial apoptosis signaling pathway in the mouse testis.

## Introduction

Asthma is a common respiratory disorder with significant morbidity and mortality worldwide. It is estimated that as many as 300 million people of all ages and all ethnic backgrounds suffer from asthma [1,2]. Characterized by airway inflammation, the disease involves multiple biological pathways including infiltration of T lymphocytes and eosinophils, increased secretion of pro-inflammatory cytokines, and reversible obstruction of bronchial tubes [3]. It is well established that the persistence of airway inflammation depends on a decrease in apoptosis of activated eosinophils and T lymphocytes [4]. In turn, surviving T cells and eosinophils cooperate to induce bronchial epithelial cell apoptosis through secretion of IFN-gamma and TNF-alpha [5,6]. Indeed, asthmatic bronchial epithelium is more susceptible to oxidant-induced apoptosis [7]. Aggregates of uncleared epithelial cell corpses (termed Creola bodies) have long been described in the sputum of asthmatics [8]. Furthermore, increased apoptosis of peripheral blood mononuclear cells was observed in patients with allergic asthma [9,10]. More importantly, it has been demonstrated that chronic asthma-induced hypoxia may result in systemic injuries such as cognitive dysfunction [11].

In the past several decades, apoptosis has been shown to play many critical roles in diverse physiological and pathological processes. Classically, it can be activated through two distinct pathways, the death receptor pathway (extrinsic) and the mitochondrial (intrinsic) pathway. During the processes of embryonic development and cell differentiation, the mitochondrial apoptosis pathway can be activated by multiple mechanisms. Similarly, it was demonstrated that the intrinsic apoptosis pathway is also activated during spermatogenesis in the testis [12]. It has been documented that apoptosis plays important roles in the germ cell differentiation, sperm maturation and survival. The process of programmed cell death is vital in the testis considering the need to eliminate sperm cells with genetic defects [13]. However, the accurate control of germ cell apoptosis during spermatogenesis is of special importance for the host, since over-activation of the pathway would decrease the sperm count and activity. Altered expression of the apoptosis-related genes was often seen in sub-fertile men and low-motility sperm. Furthermore, hazard factors and stresses such as heat and hypoxia can both induce a reduction in sperm count and can be related to the increase in germ cell apoptosis [14]. Interestingly, increased activation of the hypoxia response pathway has been noted in patients with severe asthma [15]. However, the relationship between elevated hypoxia response and apoptosis is yet to be defined in the testis [14].

A recent study demonstrates that time to pregnancy is prolonged in asthmatic females, especially in women with moderate to severe asthma due to unknown reasons [16]. However, it remains to be determined whether asthma or severe asthma has a negative effect on the male fertility. In this study, the potential relationship between asthma and sperm generation was evaluated in the mouse testis using a well-established animal model for asthma. Furthermore, the activation status of apoptosis pathways was examined in mRNA, protein and enzymatic levels. Intriguingly, we found that both sperm count and motility in asthma mice were significantly reduced in comparison with control group. Further studies showed that the Bcl-2-caspase-9 apoptosis pathway was significantly activated in the testis of asthmatic mice.

## Materials and Methods

### Mice

Mice were handled in accordance with the guidelines of the Ethics Committee of Weifang People’s Hospital (Approval Number: 2014–001) and all experiments were carried out in conformity to the Guiding Principles for the Care and Use of Laboratory Animals (NIH publication No.85-23, revised 1985). BALB/c mice of male, 6 to 8 weeks old (28–32 g, purchased from Vital River Laboratories, Beijing, China), were housed in specific pathogen-free conditions at Weifang Medical University. The mice were kept in an air-conditioned room with temperatures between 20–25°C and 50–70% humidity and acclimated for 2 weeks before experiment.

### Model of asthma

In order to generate the acute asthma model, acclimated mice were treated according to previously reported methods [17]. Briefly, mice were randomly divided into two groups, the experimental group (n = 5) and control group (n = 5). Model mice were sensitized with 20 μg of OVA (grade V, ≥98% pure; Sigma, St. Louis, Missouri) and 1 mg aluminum hydroxide (Thermo Fisher Scientific Inc.) in 0.2 mL physiological saline by intra-peritoneal (i.p.) injection on day 0, 14 and 21. Correspondingly, the control group mice were injected with 0.2 mL physiological saline. On day 28, 29, 30, 31, 32, 33 and 34, mice of the experiment group were challenged with an aerosol of OVA (1%, 50 min) using an ultrasonic nebulizer (PARI BOYN, BeiJing), while control mice were challenged with inhaled physiological saline. All mice were sacrificed 24 hours after the last challenge, and testicular tissues were harvested for further experiment.

### Tissue preparation

Each mouse was weighed before execution by CO_2_ asphyxiation. Immediately after being removed and weighed, the testes were frozen and stored in liquid nitrogen for real-time qPCR and western blotting.

### Sperm evaluation

As previously described [18], in order to measure the sperm count and motility, the left epididymis of each mouse was harvested immediately after sacrifice. Then each epididymis was homogenized in 2 mL of warm (37°C) PBS of which 20 uL was used to evaluate sperm count and motility using the hemocytometer. The sperm motility was measured in terms of the motile sperm percentage in total spermatozoa.

### Histology and immunohistochemical analysis

The mice testes were removed and fixed in 4% paraformaldehyde. Paraffin-embedded sections were made and stained with hematoxylin and eosin (HE). For apoptosis analysis, the transverse sections were incubated with cleaved-caspase-3 antibody (1:100, Cell Signaling) at 4°C overnight. The apoptotic cells were stained to brown.

### RNA preparation, PCR array and real-time qPCR

For RNA purification, frozen testis was homogenized by liquid nitrogen grinding and dissolved in Buffer RTK provided in the Qiagen RNeasy Mini Kit. RNA was extracted according to the manufacturer's instructions. All RNA samples were suspended in RNase-free water provided with the RNA isolation kit. RNA samples were treated with RNase-free DNase I (Ambion) to digest any residual chromosomal DNA. The concentration and purity of the RNA was checked with an ultraviolet (UV) spectrophotometer at wavelengths of 260 nm and 280 nm.

For PCR array, equal amounts of RNA from 5 control or experiment mice were pooled and mixed well before cDNA synthesis. Reverse transcription was carried out using PrimeScript RT Master Mix (Takara) following the manufacturer's instructions. Real-time qPCR for PCR array analysis was performed in the StepOnePlus Real-Time qPCR System (Applied Biosystems) with the SYBR Green Real-Time PCR Master Mix (Target Bio) according to the manufacturer's instructions. Duplicate plates were tested for each condition and were compared to assess reproducibility of the results. The threshold cycle (Ct) for each well was calculated using the instrument's software, and the melting-curve program was immediately run after the cycling program. The amplification efficiency of all primers was tested to be between 1.90 and 2.10. Data analysis was run by the ΔΔCt method.

**Fig 1:**
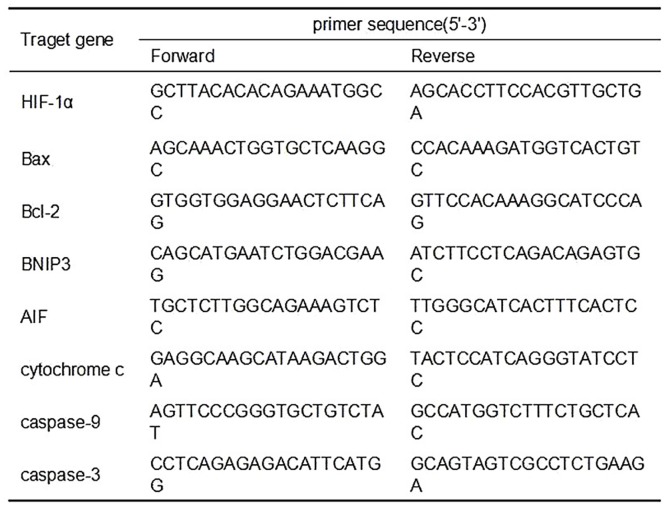
Real-time PCR primers used in the real-time qPCR assays. The forward and reverse primer sequences were listed in this form. HIF-1α, hypoxia inducible factor-1α; AIF, apoptosis-inducing factor.

For confirmation of differentially expressed genes by qPCR, equal amounts of RNA samples from the 10 mouse testes were aliquoted individually and then subjected to cDNA synthesis respectively. The mRNA expression levels of HIF-1α, Bax, Bcl-2, BNIP3, AIF, cytochrome c, caspase-9 and caspase-3 were analyzed by real-time quantitative PCR using the StepOnePlus Real-Time qPCR System (Applied Biosystems). All values were normalized to the level of glyceraldehyde-3-phosphate dehydrogenase (GAPDH) mRNA. The primers used were listed in Fig 1.

### Protein extraction and western bloting

Briefly, frozen testis were homogenized in RIPA buffer supplemented with protease inhibitors using the liquid nitrogen grinding, followed by incubation on ice for 10 min. The samples were centrifuged thoroughly to obtain protein supernatants. The protein concentrations were determined using a BCA Protein Assay Kit (Pierce). Twenty mg protein for each sample were resolved on 12% Bis-Tris or 4–12% gradient Bis/Tris gels (Life Technologies) and then transferred to PVDF membranes (Millipore). After blocking in 10% skim milk, the immunoblotting membrane was probed with indicated antibodies and visualized by ECL kit (Pierce). Lastly, images of indicated protein bands were recorded on the BioMax film (Kodak), and quantification was conducted by using Image J software (Bio-Rad). Primary antibodies used in the study were diluted as follows: anti-Bcl-2 (1:500, Santa Cruz Biotechnology), anti-PARP (1:1000, Santa Cruz Biotechnology), anti-Bax (1:500, Santa Cruz Biotechnology), anti-cytochrome c (1:500, Santa Cruz Biotechnology), anti-HIF-1α (1:500, Santa Cruz Biotechnology), anti-AIF (1:1000, Santa Cruz Biotechnology), anti-GAPDH (1: 20000, Abcam), and anti-cleaved caspase-3 (1: 1000, Cell Signaling).

### DNA ladder

DNA ladder assay for apoptosis detection was conducted following a recently updated protocol [19]. After DNA purification and concentration determination, equal amounts of genomic DNA were subjected to agarose gel electrophoresis.

### Determination of caspase-9 and 3/7 enzymatic activity

The activity of caspase-9 and caspase-3/7 was measured by Caspase-Glo-9 assay kit and Caspase-Glo-3/7 assay kit, respectively, according to the manufacturer's instructions (Promega). Briefly, protein concentration of the testis homogenates was determined for each mouse and equal amounts of the homogenates were subjected to the enzymatic assay following the standard protocol.

### Statistical analysis

All quantified data were expressed as means ± standard deviation (SD). Statistical analysis was performed using the SPSS statistical software package, version 17.0 (SPSS Inc, Chicago, IL, USA). Significance was set at the level of P < 0.05.

## Results

### Asthmatic mice showed reduced body and testis weight

**Fig 2:**
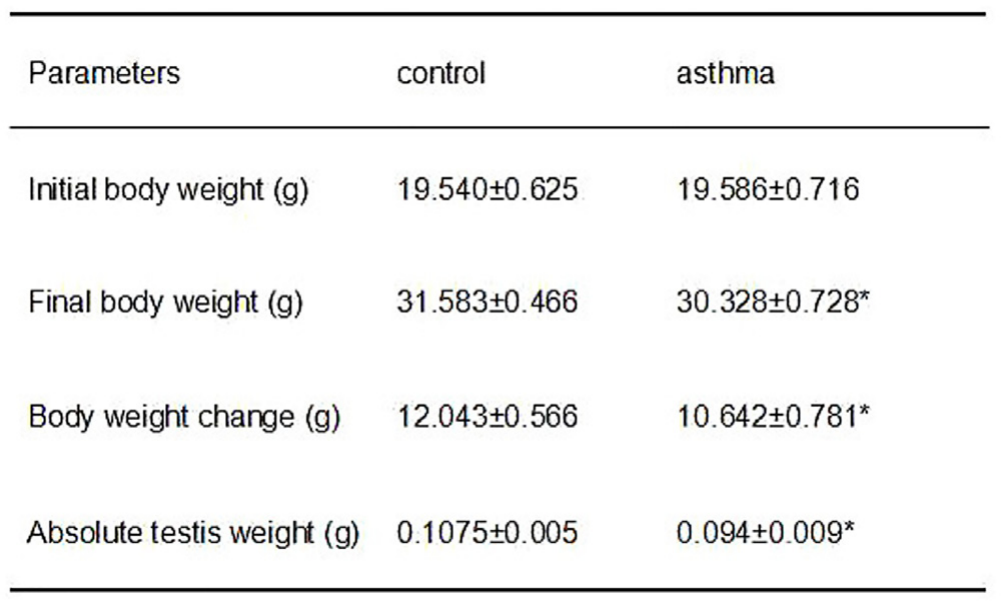
Comparison of body weight and absolute testis weight between the control and asthmatic mice. Parameters we observed including initial body weight, final body weight, body weight change and absolute testis weight. * indicates a significant difference (P<0.05), compared with the control group.

Mice rendered allergic to OVA have been widely studied, and they serve as a prototypic asthma pathogenesis model [20]. As shown in Fig 2, after the aerosol challenge for 7 days, both body weight and absolute testis weight of OVA treated mice were significantly lower than that of the control group (P<0.05). Also, the average body weight increase of asthmatic group was much lower than that of the control mice. Nevertheless, it was observed that many inflammatory cells infiltrated into the asthmatic lung (S1 Fig). These data suggest that OVA challenge may have a negative role in the maturation and development of the mice. This is consistent with previous report that physical development was altered in children and adolescents with bronchial asthma [21].

### Asthma reduced the sperm count and motility

**Fig 3:**
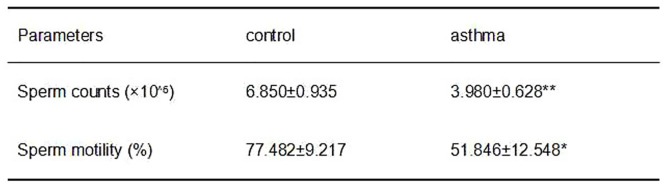
Comparison of sperm count and motility between the control and asthmatic mice. *indicates a significant difference (P<0.05), compared with the control group. **indicates a very significant difference (P<0.01), compared with the control group.

Since physical development and testis weight was altered in the asthmatic mice, we further sought to determine whether asthma could decrease sperm count and motility in the male mice. Intriguingly, results from hemocytometer measurements showed that both sperm count and motility in the asthma group were significantly lower than those in the control group (P<0.05; see Fig 3). It is worth noting that sperm count of asthmatic mice was only 58.1% of the control group, while the testis weight of asthmatic group was over 87.4% of the control mice. This suggests that apoptosis might occur more frequently in the asthmatic mice, which is further supported by the observation that sperm motility in the asthmatic mice is much lower in comparison with the control ones.

### Increased apoptosis was observed in the testis of asthmatic mice

**Fig 4:**
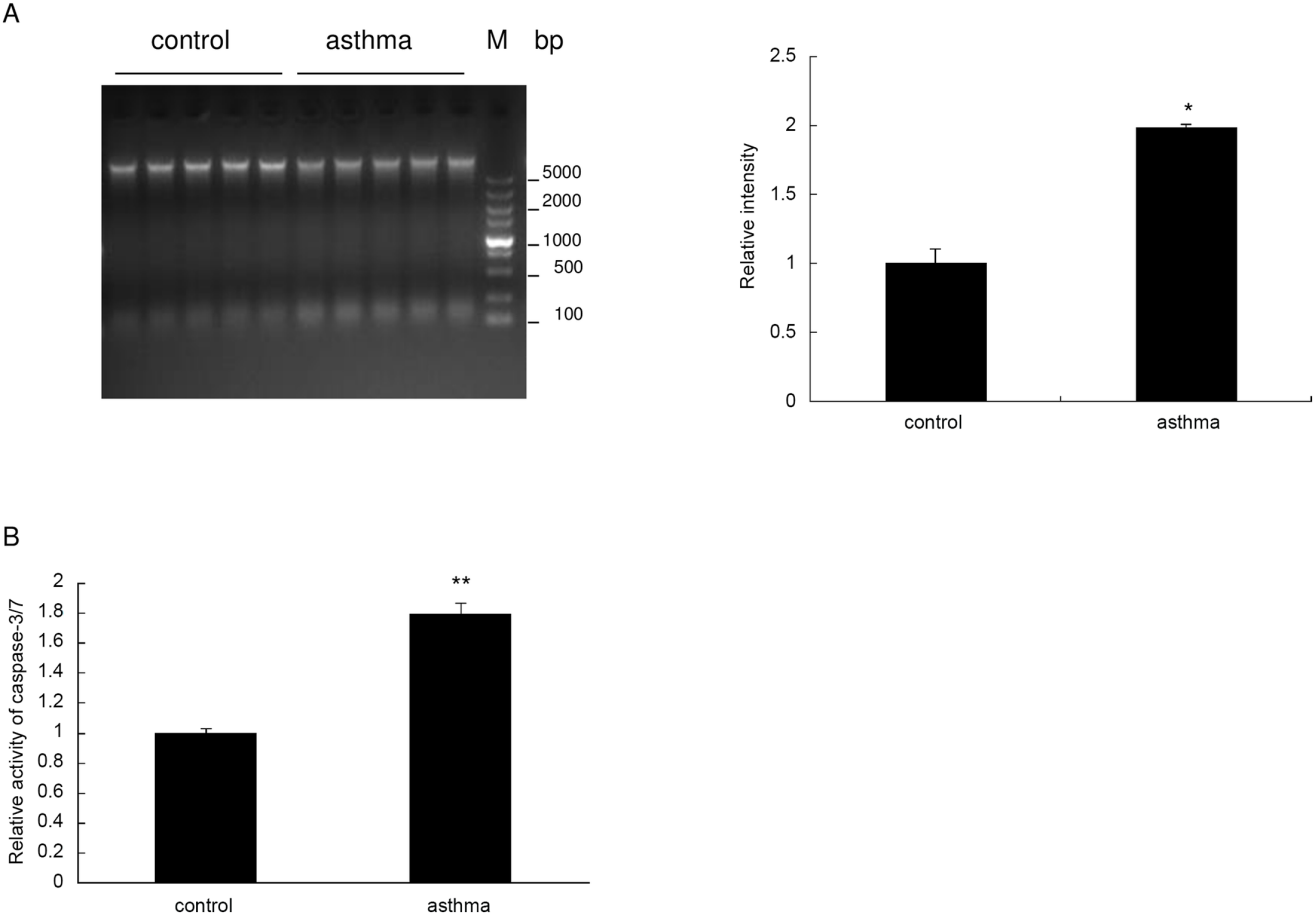
Apoptosis is enhanced in the asthmatic mouse testis. (A) A DNA ladder assay was conducted for the 10 testis samples from control and asthma group (left panel). Quantification of the bands with lower molecular weight (about 150bp) in the left panel was done by using Image J software (right panel). (B) Enzymatic activity of caspase-3/7 was measured for the testis homogenates using Caspase-Glo-3/7 assay kit, and the relative ratio was expressed in the bar graph. *indicates a significant difference (P<0.05), compared with the control group. **indicates a very significant difference (P<0.01), compared with the control group.

To see if apoptosis increases in the testis of asthmatic mice in a direct manner, we conducted a DNA ladder assay using the homogenates of the testis. Firstly, whole genomic DNA was purified according to the standard protocol. Then, equal amounts of DNA from each sample were subjected to agarose electrophoresis. It was observed that fragmentation increased markedly in the DNA of asthmatic testis compared with the control mice (Fig 4A). Consistently, as determined by the Caspase-Glo 3/7 assay kit, testis homogenates from the OVA treated mice showed a nearly 2-fold increase in caspase-3/7 activity compared with controls (Fig 4B). Taken together, these results strongly suggest that apoptosis was significantly increased in the testis of asthmatic mice.

### Histopathological changes in asthmatic mice

**Fig 5:**
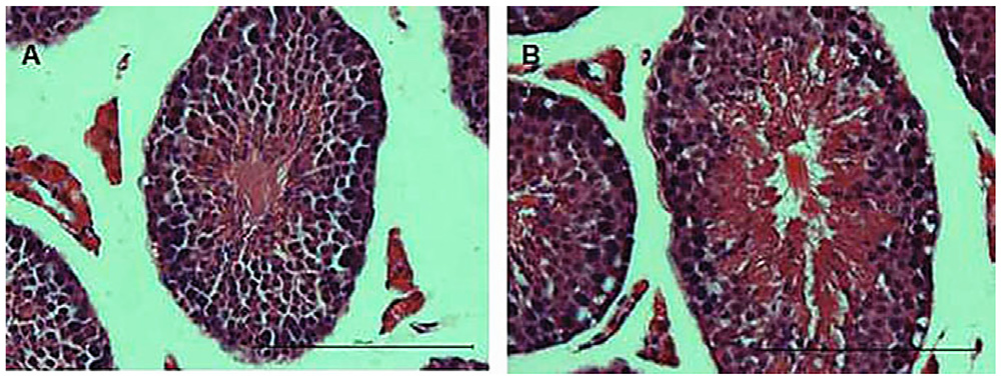
Histological changes in the testis. The transverse sections of the testis were stained with hematoxylin and eosin (HE). (A) Control group; (B) Asthma group.

Next, we investigated the potential histological changes in the mouse testis. Histological examination of the control mice showed a normal process of spermatogenesis with a regular arrangement of spermatogenic epithelium in the seminiferous tubules (Fig 5A). In contrast, the asthmatic group showed various testicular changes including the loss, derangement and sloughing of the spermatogenic cells, as well as vacuolization in the cytoplasm of Sertoli cells (Fig 5B).

### Asthma Induced the Activation of Germ Cell Apoptosis

**Fig 6:**
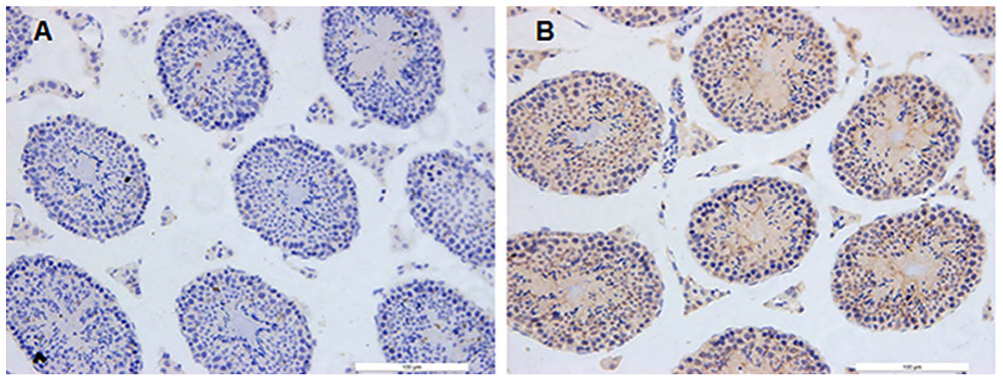
Immunohistochemisty assay for the testis. The transverse sections of the testis were stained for cleaved caspase-3. (A) Control group; (B) Asthma group.

To further identify the potential apoptotic cell types, immunohistochemistry assay was conducted using the anti-cleaved-caspase-3 antibody. As shown in Fig 6, significant increase in the cleaved caspase-3 staining was identified in the spermatogonia, spermatocytes, Sertoli cells and Leydig cells of asthma group.

### Altered mRNA level of apoptosis associated genes

**Fig 7:**
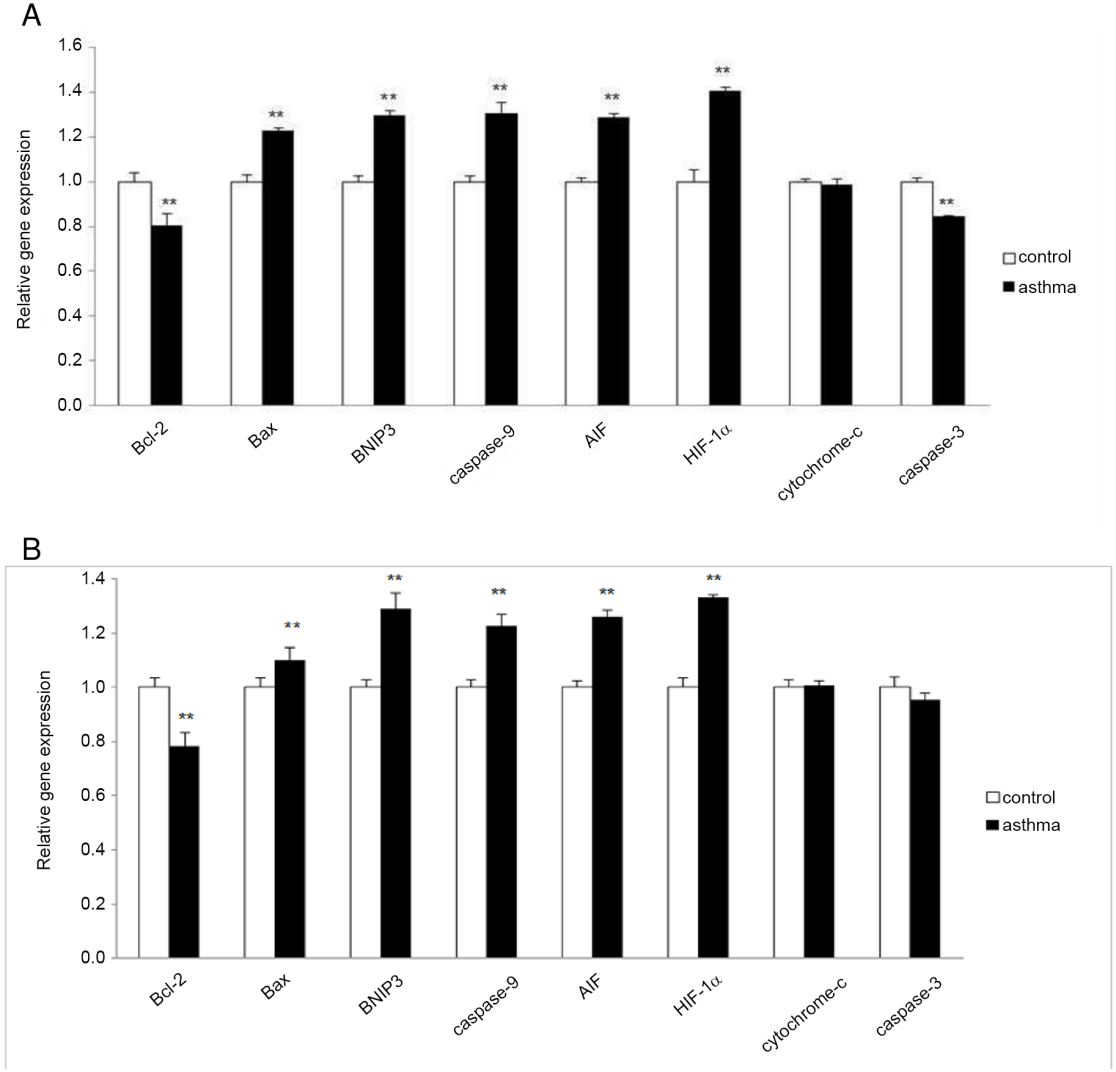
Relative mRNA level of several apoptosis-related genes. (A) Equal amounts of RNA from 5 control or experiment mice were mixed well and split into two groups before cDNA synthesis. (B) Equal amounts of RNA samples from the 10 mouse testes were aliquot individually and then subjected to cDNA synthesis respectively. Transcriptional expression of indicated genes was measured by real-time qPCR. Data were presented as means ± SD (n = 5). ** indicates a very significant difference (P<0.01), compared with the control group.

To further investigate the molecular mechanism underlying asthma-induced apoptosis in the testis, the PCR array analysis that was performed focused on a panel of hypoxia- and apoptosis-related genes. Here, the transcriptional expression of in total 120 key genes involved in the programmed cell death or hypoxia-signaling pathway was evaluated using the pooled control or asthma samples. Results showed that in the testis of OVA-induced asthmatic mice, there was a differential expression of 7 genes in comparison to unstimulated counterparts, including Bcl-2, Bax, BNIP3, caspase-9, caspase-3, AIF and HIF-1α (Fig 7A). Specific primers of these target genes were designed and RT-qPCR was carried out to confirm the observation using the individual cDNA samples of the 10 mouse testes. Consistent with the PCR array data, the transcriptional expression of Bcl-2, an anti-apoptosis factor, was markedly decreased in the testis of asthma mice, while the mRNA transcription of pro-apoptosis genes including Bax, BNIP3, caspase-9 and AIF were enhanced significantly in asthmatic testis (Fig 7B). As expected, the expression of HIF-1α was shown to be upregulated in the testis of mouse model of asthma. However, RT-qPCR demonstrated that there is no significant difference in the mRNA level of caspase-3 between the two groups (Fig 7B). As a negative control, the mRNA expression of cytochrome c was not altered in the testis of asthmatic mouse model compared with the control group (Fig 7).

### Protein level of the apoptosis associated genes

**Fig 8:**
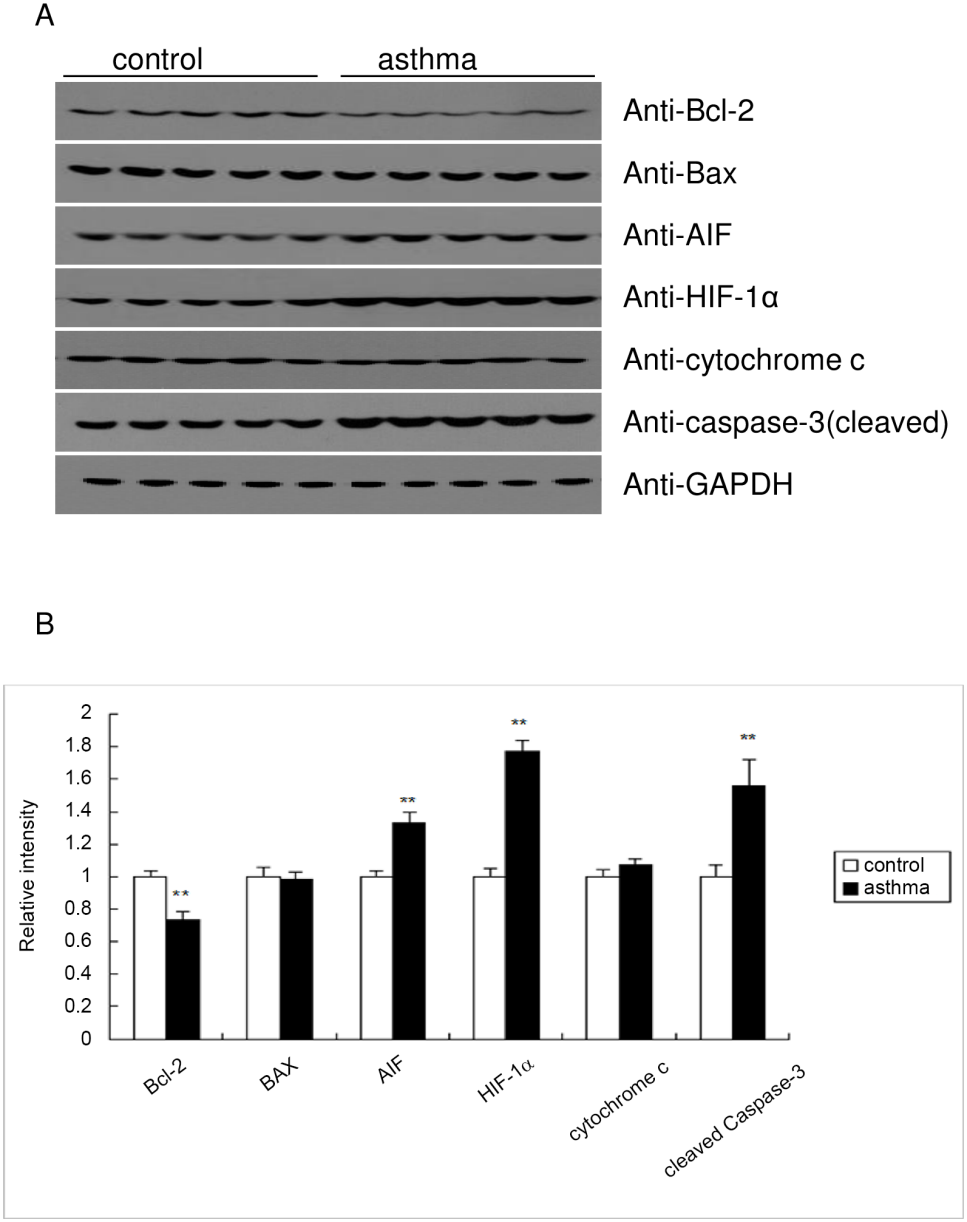
Western blot analysis of the apoptosis related proteins. (A) Equal amounts of the protein homogenate from the testis tissue were immunoblotted with the indicated antibodies. (B) Quantification of the protein expression level in (A) was performed using Image J software.

Next, we went on to examine whether asthma could regulate the protein expression of apoptosis related genes in the mouse testis. Consistent with the RT-qPCR results, protein level of the anti-apoptosis gene Bcl-2 was significantly impaired in the testis of asthma mice, while the expression of AIF and HIF-1α was significantly induced in the asthmatic testis (Fig 8). In contrast, the protein level of Bax and cytochrome c was not altered by asthma (Fig 8). Consistent with the caspase-3/7 enzymatic activity (Fig 4), the cleaved form of caspase-3 was significantly induced in the asthmatic mice (Fig 8), suggesting the activation of the caspase pathway of apoptosis in the mouse model of asthma.

### Asthma activates caspase-9 in the mouse testicular tissue

**Fig 9:**
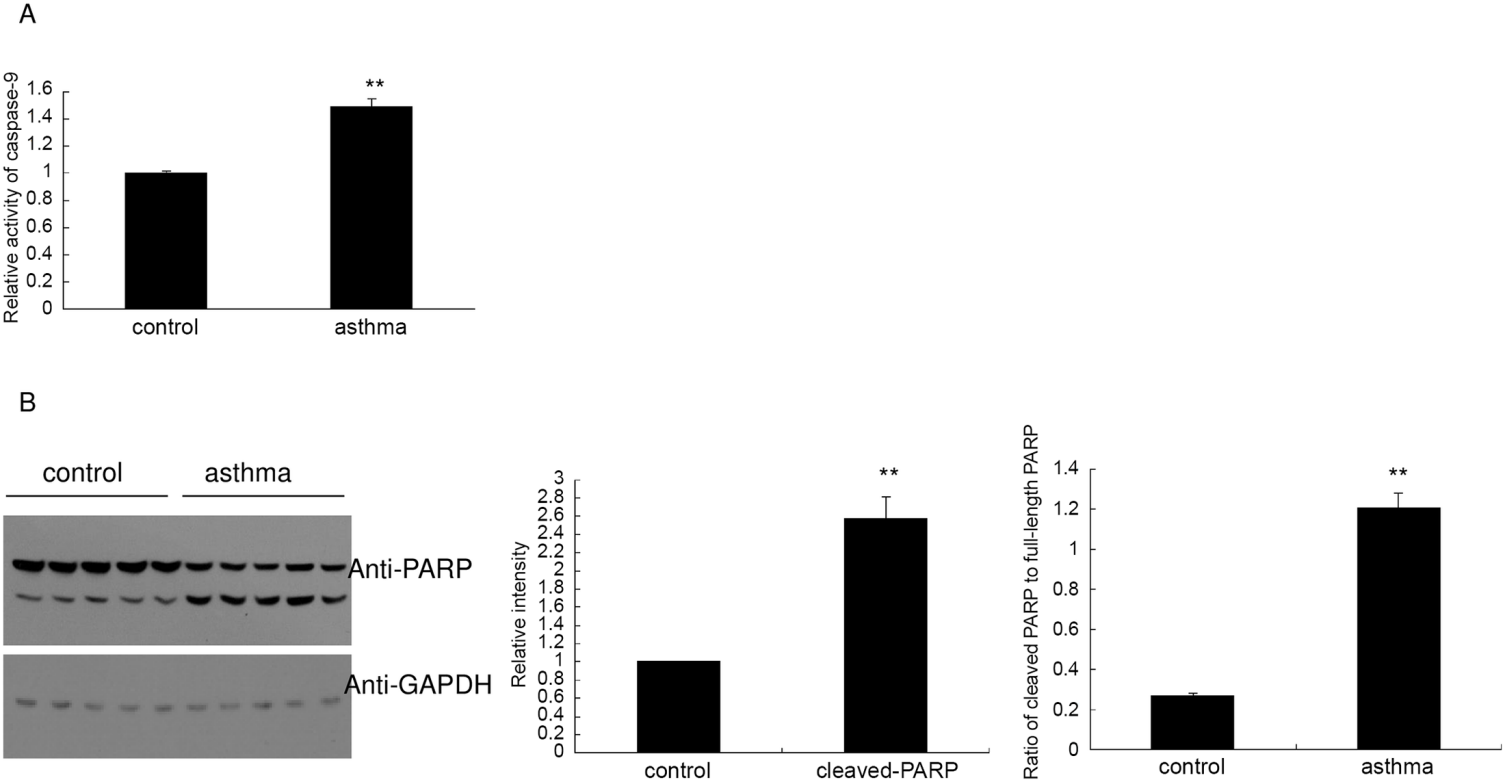
Asthma activates the proteinase caspase-9 and enhances the cleavage of PARP in the mouse testis. (A) Enzymatic activity of caspase-9 in the testis homogenates was measured by Caspase-Glo-9 assay kit, and the relative ratio was expressed in the bar graph. (B) Western blot analysis of the PARP (full-length and cleaved isoform). The relative level of cleaved PARP was determined using Image J software as demonstrated in the middle panel. In the right panel, the ratio of cleaved PARP to full-length PARP was shown based on the intensity calculated by Image J software. Data were expressed as means ± SD. ** indicates a very significant difference (P<0.01), compared with the control group.

To further investigate the involvement of caspase apoptosis pathway in the asthmatic testis, we determined the enzymatic activity of caspase-9, an upstream protease of caspase-3 and a downstream effector of the Bcl-2 mitochondrial apoptosis pathway. As shown in Fig 9A, the overall activity of caspase-9 in the homogenates of asthmatic testis was significantly induced as compared with the control mice. Collectively, these results showed that the Bcl-2-capase-9 pathway was activated in the testis of asthmatic mice.

Finally, we examined the apoptosis levels of each testis by monitoring the apoptosis marker poly ADP ribose polymerase (PARP). Consistent with the increased cleavage of caspase-3, the protein level of cleaved PARP was significantly upregulated in the asthmatic testis (Fig 9B left and middle panel). Also, the ratio of cleaved PARP to full-length PARP was increased markedly in the asthma group (Fig 9B right panel). These results were consistent with the enhanced DNA fragmentation in the asthmatic testis, as demonstrated by the DNA ladder assay (Fig 4A).

## Discussion

Asthma may have a direct effect upon physical development in children. It is an important determinant of physical development particularly for young patients with severe asthma and without proper treatment [21]. However, the exact mechanism by which asthma affects physical development has not been fully elucidated. The possible reasons involve chronic oxygen deficiencies in diverse tissues, frequent sleeplessness, and psychological stresses caused by the disease itself, which may interfere with normal childhood development [21]. Nevertheless, little is known about the causal relationship between asthma and dysfunction of the male reproductive system either in asthmatic children or in the animal model of asthma. Here we determined the sperm count and activity in an asthmatic mouse model and found that asthma may interfere with both the number and activity of mature sperm in the animal model. Further studies showed that apoptosis plays an essential role in the process. It remains largely unknown whether the sperm activity is altered in the adolescent suffering from asthma.

In this study, it was shown that OVA-induced asthma has significant effects on the physical development of mice, including the body weight loss and decreased testis weight. These defects in physical development may directly affect the number of mature sperm cells in asthmatic testis. In addition, a more significant decrease in sperm motility and viability was also found, indicating that beyond the physical development, some causes may take part in the decreased spermatogenesis. Through DNA ladder assay and immunohistochemistry, OVA-induced apoptosis was confirmed in the testis of asthmatic mice as compared with the control group. Further mechanistic studies validated the observation and demonstrated that the caspase apoptosis pathway was activated significantly in the testis of OVA-treated mice. In fact, it has been estimated that up to 75% of the hypothetical sperm number is lost due to the programmed cell death [22], while dysregulation of the process may lead to markedly decreased sperm count or motility, thus resulting in male infertility. Many environmental stresses may cause the over-activation of the apoptosis pathway in the testis, including heat, hypoxia and a series of hazardous chemicals [23]. Under the circumstances of asthma, hypoxia may be one of the main causes of elevated apoptosis in testis, as evidenced by the induction of HIF-1α expression. In addition, it has been demonstrated that spermatogenesis may be affected by many other reasons, such as hormone levels [24]. Hence, it is possible that biological pathways other than apoptosis play roles in the sperm maturation and viability after OVA-treatment. To elucidate this hypothesis, caspase inhibitors could be applied to asthmatic mice to examine if sperm production could be rescued to the control level.

Previously, it has been demonstrated that HIF-1α expression is potentiated in the lung of asthma mouse models, as well as in BALF cells from asthmatic patients [25]. Here, we found that HIF-1α expression was also markedly induced in the asthmatic testis both in the mRNA level and protein level. As a key regulator of the hypoxia response, protein expression of HIF-1α can be induced in a variety of tissues after oxygen deprivation and form the heterodimeric transcription factor HIF-1 together with the HIF-1β [26]. In turn, the nucleus-located HIF-1 can initiate apoptosis by inducing high concentrations of pro-apoptotic proteins, such as BNIP3 [27]. Consistently, the induction of BNIP3 was identified in the asthmatic testis by PCR array. However, it was reported that anti-apoptotic proteins, such as IAP-2, may also be induced during hypoxia, thus counteracting the apoptosis process. Therefore, an intricate balance may exist between factors that induce or counteract apoptosis. In addition to BNIP3, the expression of pro-apoptosis factors Bax, AIF and caspase-9 was induced, while the level of anti-apoptosis factor Bcl-2 was decreased in the present study. Cooperatively, these proteins induce the asthmatic testis to undergo apoptosis rather than survival. Nevertheless, the upstream pathway of Bax, AIF and caspase-9 inductions and Bcl-2 inhibition remain to be defined in the asthmatic testis.

As downstream events of apoptosis, we determined the enzymatic activity of caspase-9 and caspase-3/7, together with the cleavage of caspase-3 and PARP. These results demonstrate that the caspase apoptosis pathway is markedly activated in asthmatic testis compared with the control group, which might be the reason for decreased sperm viability and mobility. Previously, it was reported that apoptosis is not mediated exclusively by caspases. Among them, Apoptosis-inducing factor (AIF) was the first identified caspase-independent cell death effector which may suffice to induce cell death. AIF may be released from the mitochondrial intermembrane space along with cytochrome c and translocate to the nucleus, where it promotes DNA fragmentation, probably through endonuclease G (EndoG) [28]. Interestingly, PCR arrays identified that the transcriptional level of apoptosis-inducing factor AIF was elevated, and western blot confirmed the increased protein expression in asthmatic testis. Therefore, caspases and caspase-independent death effectors such as AIF may cooperate to induce apoptosis in asthmatic mice. Together with the observation of Bcl-2/Bax involvement, these results collectively suggest that asthma may lead to the activation of mitochondrial pathway of apoptosis.

As revealed by the immunohistochemistry (Fig 6), a significant proportion of spermatogonia and spermatocytes undergo apoptosis, while only a minor proportion of somatic cell types such as Sertoli cells and Leydig cells is lacking. So the mRNA and protein of the somatic cell types might get enriched in the asthmatic testis and the enrichment of non-germ cells may directly alter the overall enzymatic activities of caspase-9 and caspase-3/7 [29]. Furthermore, the loss of specific cell types may distort the relative levels of gene products produced by those cells [30]. When this is the case, the increased mRNA levels might be a result of simple non-germ cell enrichment due to impaired spermatogenesis. Therefore, it might be very hard to make comparisons between the apparent levels of an mRNA species or a protein in an apoptosis testis and the levels in a control, unaffected testis. To further clarify the apoptosis pathway involved in the process, the transverse sections of testis should be subjected to immunohistochemistry for staining of the apoptosis proteins in future studies.

## Conclusions

Conclusively, the present study demonstrates that asthma could induce apoptosis in testis tissue possibly through the mitochondrial pathway, in which caspase-9, caspase-3 and PARP are significantly activated. As a result, apoptosis in asthmatic testis may ultimately lead to the infertility problems in male. To our knowledge, this is the first report that asthma could lead to the enhanced activation of mitochondrial apoptosis signaling pathway in the testis of mice.

## Supporting Information

S1 Fig Lung histology was stained by HE and imaged by light microscopy under ×50 magnifications. (A) control group. (B) asthma group.
(TIF)
