# Supplementary figures and images for: Activation of Bcl-2-Caspase-9 Apoptosis Pathway in the Testis of Asthmatic Mice
Source: PLoS One. 2016 Mar 3;11(3):e0149353. doi: 10.1371/journal.pone.0149353 (PMC4777281; doi:10.1371/journal.pone.0149353)

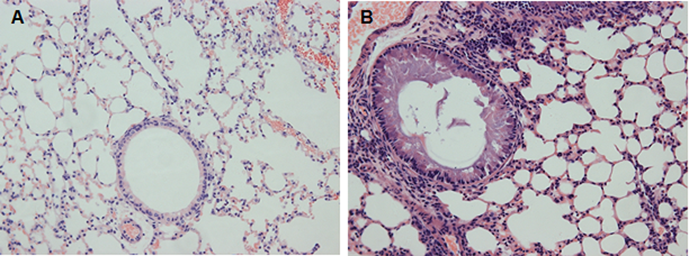

Supplement: S1 Fig — (A) control group. (B) asthma group. (TIF) [file pone.0149353.s001.tif]
